# The Wnt inhibitory factor 1 restoration in prostate cancer cells was associated with reduced tumor growth, decreased capacity of cell migration and invasion and a reversal of epithelial to mesenchymal transition

**DOI:** 10.1186/1476-4598-9-162

**Published:** 2010-06-23

**Authors:** David S Yee, Yaxiong Tang, Xuesen Li, Zhongbo Liu, Yi Guo, Samia Ghaffar, Peter McQueen, Dash Atreya, Jun Xie, Anne R Simoneau, Bang H Hoang, Xiaolin Zi

**Affiliations:** 1 Departments of Urology and Chao Family Comprehensive Cancer Center, University of California at Irvine Orange, CA 92868, USA; 2 Department of Orthopaedic Surgery and Chao Family Comprehensive Cancer Center, University of California at Irvine, Orange, CA 92868, USA; 3 Chengdu Institute of Biology, the Chinese Academy of Sciences, Chengdu, Sichuan, PR China 610041

## Abstract

**Background:**

Aberrations in the Wnt pathway have been reported to be involved in the metastasis of prostate cancer (PCa) to bone. We investigated the effect and underlying mechanism of a naturally-occurring Wnt inhibitor, WIF1, on the growth and cellular invasiveness of a bone metastatic PCa cell line, PC3.

**Results:**

The WIF1 gene promoter was hypermethylated and its expression down-regulated in the majority (7 of 8) of PCa cell lines. Restoration of WIF1 expression in PC-3 cells resulted in a decreased cell motility and invasiveness via up-regulation of epithelial markers (E-cadherin, Keratin-8 and-18), down-regulation of mesenchymal markers (N-cadherin, Fibronectin and Vimentin) and decreased activity of MMP-2 and -9. PC3 cells transfected with WIF1 consistently demonstrated reduced expression of Epithelial-to-Mesenchymal Transition (EMT) transcription factors, Slug and Twist, and a change in morphology from mesenchymal to epithelial. Moreover, WIF1 expression significantly reduced tumor growth by approximately 63% in a xenograft mouse model. This was accompanied by an increased expression of E-cadherin and Keratin-18 and a decreased expression of vimentin in tumor tissues.

**Conclusion:**

These data suggest that WIF1 regulates tumor invasion through EMT process and thus, may play an important role in controlling metastatic disease in PCa patients. Blocking Wnt signaling in PCa by WIF1 may represent a novel strategy in the future to reduce metastatic disease burden in PCa patients.

## Introduction

Prostate cancer (PCa) is the second most frequent cause of cancer-related mortality in men in the United States [1,2]. Although a significant portion of PCa is curable either by surgery or by radiotherapy when detected early [1,2], advanced PCa with metastases still presents a difficult therapeutic problem. Specifically, bone metastasis is the major cause of mortality in patients with PCa. Therefore, understanding the processes that promote metastasis of PCa could be useful in designing effective therapies for advanced PCa with bone metastasis.

The wingless-type (Wnt) pathway plays a central role in the development of many tissues and organisms. Aberrant activation of the Wnt pathway contributes to the progression of several major human cancers, including PCa [3,4]. The best-studied Wnt signaling pathway is the Wnt/β-catenin pathway, comprised of secreted Wnt ligands and cell-surface receptors called Frizzled and Lipoprotein Receptor-Related Protein 5/6 (LRP5/6) [3,4]. In the presence of ligandreceptor binding, cytosolic β-catenin is translocated into the nucleus, and forms a complex with TCF family of transcription factors to activate target genes [3,4]. In contrast, Wnt inhibition leads to decreased accumulation of cytosolic and nuclear β-catenin with consequent downregulation of Wnt-responsive genes [3,4]. An exhaustive list of Wnt target genes has been posted in "The Wnt homepage" website, which includes cell cycle regulators, metalloproteinase (MMP), CD44, Met, Jagged1, vascular endothelial growth factor, etc. [5]. This list indicates that the Wnt pathway participates in not only cell proliferation, but also cell invasion, metastasis and angiogenesis through regulation of Wnt target gene expression in a context-dependent fashion.

In addition to Wnt ligands and receptors, three classes of secreted antagonists of the Wnt pathway have been identified: secreted Frizzled-related protein (sFRP) family, Dickkopf (Dkk) family, and Wnt inhibitory factor 1 (WIF1) [6-9]. These antagonists can modulate Wnt signaling either by binding to Wnt ligands or by binding to the LRP5/6 co-receptor, leading to receptor endocytosis [6-9]. Initially, it was thought that activating mutations of APC or β-catenin were the dominant mechanisms of Wnt activation in cancer [10]. However, recent evidence shows that secreted Wnt antagonists (e.g. sFRP1) can suppress Wnt signaling despite the presence of these down-stream activating mutations, suggesting autocrine Wnt signaling could be involved in cancer progression [11,12]. Moreover, emerging evidence demonstrates that PCa tissues and cell lines, as well as stromal components of the prostate express Wnt ligands and receptors, thus implicating autocrine or paracrine signaling in at least a subset of prostate tumors [13-16]. As such, the use of secreted antagonists to suppress autocrine and/or paracrine Wnt signaling and its downstream targets in PCa may be a viable option for reduction of tumor burdens.

WIF1 silencing by hypermethylation and consequent Wnt signaling activation has been demonstrated in numerous cancers such as nasopharyngeal cancer [17], lung cancer [18], mesothelioma [19], breast cancer [20], urinary bladder cancer [21], renal cancer [22], osteosarcoma [23,24] and gastric cancer [25]. Compared to other secreted Wnt antagonists, WIF1 has been consistently shown to inhibit the *in vitro *and *in vivo *growth of various cancer cells [26,27]. WIF1 is down-regulated in 64% (27 of 42) of primary PCa specimens [26] and overexpression of WIF1 in PC3 cells increases the efficacy of Paclitaxel to induce apoptosis [27]. However, the mechanism of WIF1 down-regulation and the function roles of WIF1 expression in PCa remain unknown.

In addition, WIF1 has been implicated to play a role in normal prostate development [28,29]. In a study on early androgen induced prostate development, nearly all Wnts, along with WIF1 were expressed in the developing prostate, suggesting Wnt signaling as one of the major androgen regulated pathways in early prostate development [28]. In a tissue recombination experiment for studying induction of prostate formation, the overexpression of WIF1 in urogenital sinus mesenchyme inhibited prostate formation [29]. Furthermore, WIF1 was shown to be highly expressed in the developing and mature mouse skeleton and involved in osteoblast differentiation and chondrogenesis [30,31]. Targeted deletion of mouse WIF1 augmented spontaneous and radiation-induced osteosarcoma formation [23]. Together, these studies suggested that WIF1 plays an important functional role in both the prostate and bone (the most common metastasis site of PCa). Therefore, further investigation is warranted regarding WIF1 down regulation in PCa and the potential of WIF1 as a future target for novel therapies.

In this report, WIF1 expression was down-regulated in the majority of PCa cell lines via promoter hypermethylation. Ectopic expression of WIF1 in a bone-metastatic PCa cell line PC-3 resulted in up-regulation of epithelial markers (E-cadherin, Keratin-8 and-18) and down-regulation of mesenchymal makers (N-cadherin, Fibronectin and Vimentin) both *in vivo *and *in vitro*, suggesting a reversal of EMT. Moreover, WIF1 expression resulted in decreased cell motility and invasiveness in cell cultures, as well as reduced tumor growth in a PCa xenograft model.

## Materials and methods

### Plasmids, cell lines and stable transfection

PC3, 22Rv1, LNCaP and DU145 cells were obtained from American Type Culture Collection (Manassas, VA) and maintained in RPMI 1640 supplemented with 10% fetal bovine serum (FBS) and antibiotics. C4-2B (ViroMed Laboratories; Minneapolis, MN), an LNCaP subline, was grown in RPMI 1640 supplemented with 5% FBS. LAPC-4 (a gift from Dr. C. Sawyer) cells were cultured in RPMI 1640 supplemented with 10% FBS. PC3 M and PC3M-LN4 Cells were kindly provided by Dr. Curtis Pettaway (M. D. Anderson Cancer Center, Houston, TX) and were cultured in RPMI medium containing 10% FBS. Primary prostate epithelial cells (PrEC) came from Cambrex Bioscience (Walkersville, MD) and were grown in prostate epithelial basal medium. *PCDNA3.1 *Directional TOPO Expression vector was obtained from Invitrogen. An Ultimate ORF *WIF1 *clone (ID: IOH11153) was obtained from Invitrogen, subcloned into the *PCDNA3.1 *TOPO vector containing a V5 tag and verified by automated DNA sequencing using standard methods. For stable transfection, PC3 cells were plated at 1.6 × 10^5 ^per 100-mm dish. At 60% confluency, cultures were transfected with *PCDNA3.1 or WIF1 *using FuGENE 6 (Roche) according to manufacturer's instruction. After transfection, stable clones were selected with G418 (800 μg/ml) starting at 48 h post transfection and assayed for expression of the transgene by western blot and real-time RT-PCR. Pooled transfectants (to avoid cloning artifacts) are propagated and maintained in RPMI 1640 containing 10% FBS and 500 μg/mL G418.

### Luciferase and β-galactosidase assays

PC3 cells stably expressing *PCDNA3.1 *or *WIF1 *were grown in six-well plates and transiently cotransfected with 1 μg pTOPFLASH or pFOPFLASH and 0.1 μg cytomegalovirus (CMV)-β-galactosidase plasmids (Invitrogen, Carlsbad, CA) using FuGENE 6. After 24 hours of incubation, cells were harvested and the luciferase and β-galactosidase activities were measured using Bright-Glo luciferase assay system and β-galactosidase enzyme assay system (Promega, Madison, WI). The relative luciferase unit for each transfection was adjusted by β-galactosidase activity in the same sample.

### Protein extraction, conditioned medium, and Western blotting

For extraction of membrane and cytosolic proteins [32], cells were collected in TES suspension buffer and homogenized on ice. The cytosolic fraction was recovered by ultracentrifugation at 100,000 × g. The membrane-enriched pellet was solubilized in solubilization buffer. For extraction of total proteins, cells were lysed in radioimmunoprecipitation assay buffer. Conditioned media were prepared using serum-free RPMI by culturing PC3 cells stably transfected with vector control or WIF1 at 70% confluence for 48 hours and concentrated 40 times by Centricon (Millipore, Bedford, MA). Clarified protein lysates (20-80 μg) or concentrated conditioned medium were electrophoretically resolved on denaturing SDS-polyacrylamide gel (8-16%), transferred to nitrocellulose membranes, and probed with antibodies against β-catenin (Upstate Biotechnology, Charlottesville, VA), E-cadherin and N-cadherin (BD Biosciences, San Diego, CA), keratin-8, keratin-18, fibronectin, and vimentin (Lab Vision, Fremont, CA), Phospho-AKT, AKT, GSK-3β and phospho-GSK-3β (Cell Signaling, Beverly, MA), c-myc (Calbiochem, San Diego, CA), cyclin D1, Slug, Twist, WIF1, and β-actin (Santa Cruz Biotechnology, Santa Cruz, CA). Proteins were revealed using secondary antibodies and visualized by an enhanced chemiluminescence detection system (Amersham Bioscience, Piscataway, NJ).

### Immunocytofluorescence assay

Cells were cultured in chamber slides (Lab-Tek). After methanol fixation and permeabilization with Triton X-100, cells were incubated with an anti-E-cadherin antibody (BD Biosciences) and then with an Alexa 488-conjugated secondary antibody (Molecular Probes, Inc., Eugene, OR). Localization of E-cadherin was analyzed under confocal microscopy (Zeiss, Thornwood, NY) using the 488 nm excitation wavelengths of the laser.

### RT-PCR and RT-real time-PCR

Total RNA was isolated from PC3, 22Rv1, LNCaP, C4-2B, PrEC, LAPC-4, PC3 M, PC3M-LN4, DU145, *PcDNA3.1*/PC-3 and *WIF1*/PC-3 cells using the RNAazol B method as described [33]. RNA was treated with ribonuclease-free DNaseI to remove any contaminating DNA. Reverse transcription of 1 μg total RNA was performed using the Reverse Transcription System (Promega) with oligo-dT primers. Real-time RT-PCR was performed as previously described [32] using a MyiQ real-time thermocycler (Bio-Rad). The PCR condition was as follows: 95°C for 5 minutes, 40 cycles of 30 seconds at 95°C, 30 seconds at 68°C, 60 seconds at 72°C. Relative quantitative fold change compared to control was calculated using the comparative Ct method [32]. Data were analyzed by using the comparative Ct method [32], where Ct is the cycle number at which fluorescence first exceeds the threshold. The Ct values from each sample were obtained by subtracting the values for *β-actin *Ct from the *WIF1, E-cadherin, Twist, or Slug *Ct value. The variation of *β-actin *Ct values is <0.5 among different samples. One difference of Ct value represents a 2-fold difference in the level of mRNA. Specificity of resulting PCR products was confirmed by melting curves. C_t _is the cycle number at which fluorescence intensity first exceeds the threshold level. Δ C_t _is C_t _(target gene) - C_t _(actin). Gene specific primer pairs (including product size) are available upon request. Specificity of amplification products were checked by melting curve analysis and agarose gel electrophoresis.

### Matrigel invasion assay

To assay cell motility, 2.5 × 10^4 ^cells per well in serum-free RPMI were placed in the upper chamber. RPMI plus 10% FBS was placed in the lower chamber as a source of chemoattractant. Cells were allowed to migrate through a porous, uncoated membrane (BD Biosciences) for 24 hours at 37°C. Nonmigratory cells in the upper chamber then were removed with a cotton-tip applicator. Migrated cells on the lower surface were fixed with methanol and stained with hematoxylin. The number of migrating cells was determined by counting 10 high-power fields (×100) on each membrane and calculated as mean number of cells per field. For invasion assays, 24-well invasion chamber system (BD Biosciences) was used. Viable cells (2.5 × 10^4^/well) in serum-free RPMI were seeded in the upper chamber coated with Matrigel. RPMI plus 10% FBS was placed in the bottom well. Incubation was carried out for 48 hours at 37°C. The membrane was processed as described for the motility assay. An invasion index, corrected for cell motility, was calculated as follows:

All of the cell lines were assayed in triplicate for each experiment, and each experiment was repeated thrice.

### Gelatin zymography

Samples were applied to nondenaturing 10% polyacrylamide gels containing 1 mg/mL gelatin. After electrophoresis, the gels were washed with 2.5% Triton X-100, incubated overnight at 37°C in zymography buffer, and stained with Coomassie brilliant blue. Gelatinolytic activity was visualized as clear areas of lysis in the gel.

### Motility assay

Motility was assessed with a scratch assay to measure two-dimensional cellular movement. *PcDNA3.1 *vector control and *WIF1*-transfected PC3 cells were cultured to confluence in 24-well plates. A scratch was made on the monolayer using a sterile pipette tip. The monolayer was washed with migration assay buffer consisting of serum-free medium plus 0.1% BSA. At the initiation of the experiment, a digital image of the scratch wound was taken at 10× magnification. At 30 hours, the same region was imaged again. The width of the scratch wounds was measured in Photoshop7.0 (Adobe). The relative fold change of the scratch wound width (%) at 30 hour after introduction of the scratch wound compared to the control was calculated as the average of 5 fields (40× magnification).

### Methylation studies

Genomic DNA was obtained from PCa cell lines including PC3, 22Rv1, LNCaP, C4-2B, LAPC-4, PC3 M, PC3M-LN4, and DU145 as well as from PrEC cells using Blood & cell culture DNA mini kit (Qiagen). In order to quantify DNA methylation, the EZ DNA Methylation-Gold Kit (Zymo Research) was used. Briefly, 20 μl (2 μg) of genomic DNA was diluted in C to T conversion reagent (bisulfite). The DNA was denatured and converted at 98°C for 10 minutes and 64°C for 2.5 hours. Bisulfite-treated DNA was then cleaned and desulphonated using the M-binding buffer, followed by wash buffer, and desulphonation buffer. The bisulfite modified DNA was amplified by PCR using a pair of methylation specific primers (MSP, forward: 5'-GGGCGTTTTATTGGGCG TAT-3'£» reverse: 5'-AAACCAACAATCAACGAAC-3') and un-methylation specific primers (UMSP, forward: 5'GGGTGTTTTATTGGGTGTAT-3'; reverse: 5'-AAACCAACAATCAACAAAAC-3).

### In vivo tumor model

NCR-nu/nu (nude) mice were obtained from Taconic (Germantown, NY). Cells from each stable line were concentrated to 2 × 10^6^ per 200 μL and injected s.c. into the left flank of each mouse. Once xenografts became established, their sizes were measured every 3 days. The tumor volume was calculated by the formula: 0.5236L1(L2)2, where L1 is the long axis and L2 is the short axis of the tumor. All of the animal studies were approved by the Institutional Animal Care and Use Committee at University of California (Irvine, CA).

### Immunohistochemistry

Tumor tissue slides were de-paraffinized and dehydrated using Slide Brite (Sasco Chemical Group, Inc.). Antigen was retrieved using 0.05 M Glycine-HCL buffer, pH 3.5, containing 0.01% (w/v) EDTA, at 95°C for 20 min and stained with an antibody against human E-cadherin (1:500), Keratin-18 (1:500) and Vimentin (1:500). Staining was visualized with diaminobenzadine using the Cell and Tissue Staining kit (R&D Systems). The immunostaining was scored as positive or negative for E-cadherin, Keratin-18 and Vimentin by a pathologist experienced in immunohistochemistry of human tissue sections.

### Statistics

Comparisons of cell density, number of colonies, invasion index, relative levels of mRNA expression, the width of the scratch wounds and relative levels of protein expression between the different transfections were conducted using Student's t test. For tumor growth experiments, repeated-measures ANOVA was used to examine the differences in tumor sizes among different transfections, time points, and transfection-time interactions. Additional post-test was done to examine the differences in tumor sizes between vector control and other transfections at each time point by using conservative Bonferroni method. All statistical tests were two sided. P < 0.05 was considered statistically significant.

## Results

### The absence of WIF1 mRNA expression in the majority of PCa cell lines is associated with WIF1 promoter hypermethylation

**Figure 1:**
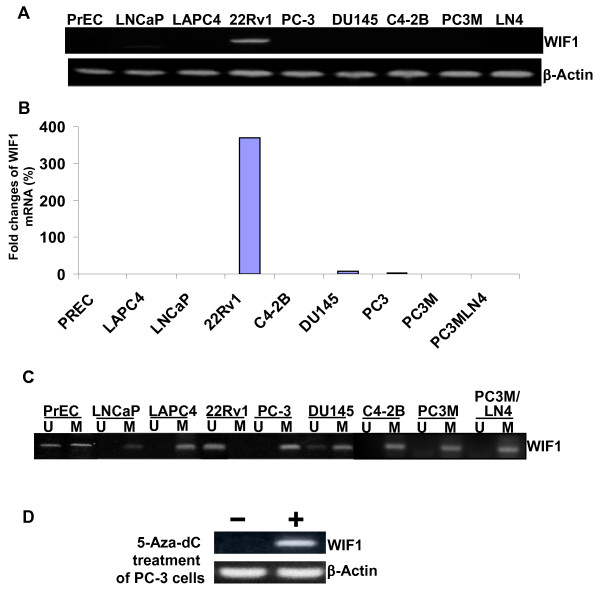
**The loss of WIF1 expression in the majority of human PCa cell lines is associated with its promoter hypermethylation**. **A&B**, the expression of WIF1 mRNA in eight PCa cell lines (LNCaP, LAPC4, 22Rv1, PC3, DU145, C4-2B, PC3 M, PC3-LN4) and PrEC were detected by regular RT-PCR and quantified by real-time RT-PCR. Experiments were replicated thrice. **C**, WIF1 promoter hypermethylation is evident in seven of eight PCa cell lines. The methylation status of WIF-1 promoter was determined by the disulfite treatment of cell lines followed by methylation-specific PCR; M, methylation-specific primers; UM, unmethylation-specific primers. ***D***, WIF1 expression in PC3 was reactivated by a demethylation agent 5-Aza-dC. After PC3 cells were treated with 5-Aza-dC (5 μmol/L) for 72 h, RT-PCR was done to detect the WIF1 mRNA expression.

WIF1 mRNA and protein expression was reported to be down-regulated in PCa tissues [26]. However, the mechanism of WIF1 down-regulation in PCa cells *per se *has not been reported yet. We show here that WIF1 mRNA expression is absent in the majority of PCa cell lines, as well as in normal prostate epithelial cells (Figure 1A &1B). Only 22Rv1 cell line has strong WIF1 mRNA expression (Figure 1A &1B). In addition, Figure 1C shows that all PCa cell lines and normal prostate epithelial cells with loss of WIF1 expression exhibit a strong methylation status in its promoter, whereas the promoter methylation of WIF1 in 22Rv1 cell line was not detected (Figure 1C). Furthermore, treatment of PC-3 cells with 1 μM 5-azacytidine for 72 hours induced the re-expression of WIF1 mRNA (Figure 1D). Together, these results suggest that the absence of WIF1 mRNA expression in the majority of PCa cell lines is due to WIF1 promoter hypermethylation.

### WIF1 inhibits the canonical Wnt pathway

**Figure 2:**
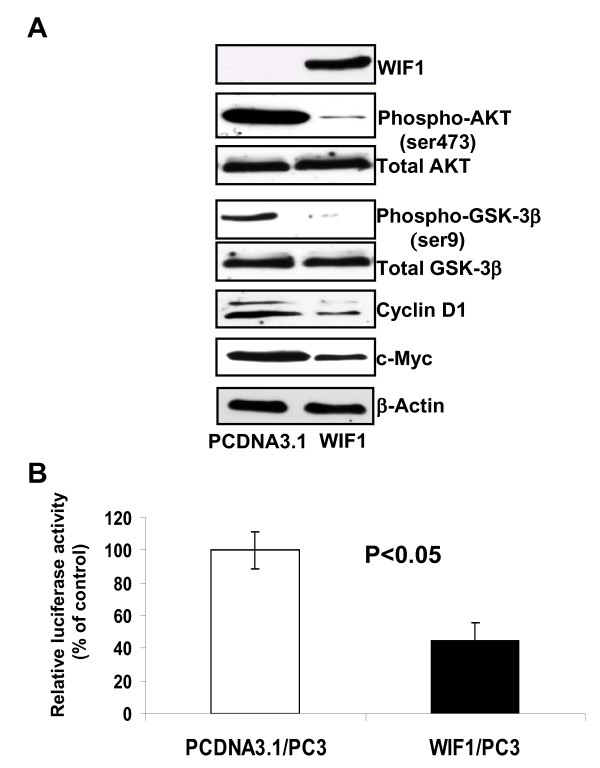
**Ectopic expression of WIF1 in PC3 cells inhibits AKT and GSK-3β, phosphorylation and LEF1/TCF transcriptional activity, leading to down-regulation of the expression of Wnt target genes: cyclin D1 and c-Myc**. **A**, Western blot analysis of expression of WIF1, phospho-AKT, total AKT, Phospho-GSK-3β, total GSK-3β, cyclin D1 and c-Myc was shown by a representative blot from three independent experiments. **B**, WIF1 transfection reduced TCF4 transcriptional activity in PC3 cells compared with vector control-transfected cells, measured by the TOPFLASH reporter assay and adjusted by transfection efficiency.

Bone metastases occur in approximately 70% of patients with advanced PCa [34]. Since PC3 cell line was derived from a bone metastatic PCa specimen, this cell line was selected for further analysis of WIF1 mediated Wnt signaling blockade. We confirmed the protein expression of the WIF1 transgene tagged by V5 in stable PC3 cell line by Western blot analysis using an anti-V5 antibody (Figure 2A). Figure 2A also shows that WIF1 expression caused a decreased expression of both phospho-GSK3β at serine-9 and -AKT at serine-473 without changing the levels of their total protein. In addition, WIF1 expression in PC3 cells decreased the protein levels of two common Wnt target genes: Cycline D1 and c-Myc. Furthermore, to examine the inhibition of canonical Wnt activity by WIF1, LEF-1/TCF transcriptional activity was assessed by TOPFLASH luciferase reporter assay in vector and WIF1 transfected PC3 cell lines. Adjustment of transfection efficiency was performed by co-transfection of a β-galactosidase expression vector. Compared to controls, WIF1 reduced LEF-1/TCF transcriptional activity by approximately 56% (Figure. 2B, Student's t test; P < 0.05). Together, these results indicate WIF1 expression inhibits canonical Wnt activity in PC3 cells.

### WIF1 expression induces a morphological transformation of PC3 cell from a fibroblastic to an epithelial appearance via up-regulation of epithelial markers and down-regulation of mesenchymal markers in PC3 cells

**Figure 3:**
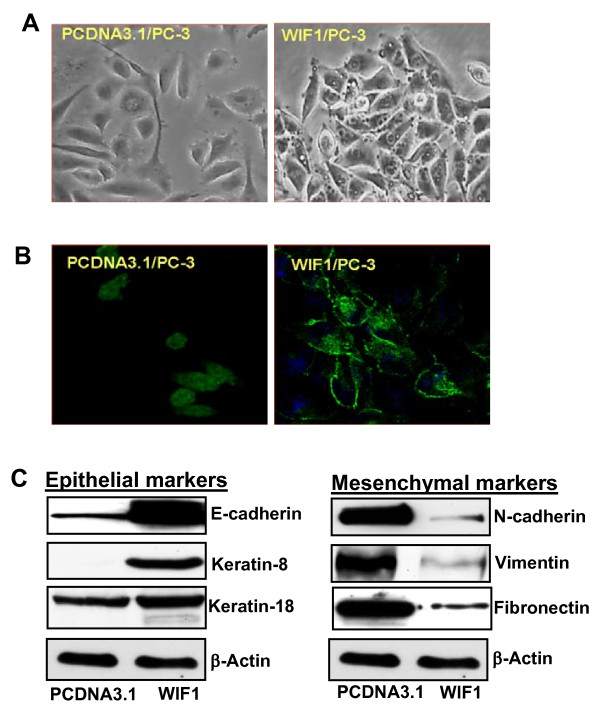
**Ectopic expression of WIF1 in PC3 cells results in a morphology change from a fibroblastic to an epithelial appearance, accompanied by up-regulating the expression of epithelial markers and down-regulating the expression of mesenchymal markers**. **A**, representative photograph of transfected cells at 60% confluence was taken under an inverted phase-contrast light microscope at ×200 magnification. **B**, the transfectants were stained with anti-E-cadherin and Alex 488-conjugated secondary antibodies. Representative photographs were taken under a confocal microscope (Zeiss) at ×400 magnification. **C**, Western blot analysis of expression of E-cadherin, keratin-8, keratin-18, N-cadherin, vimentin, fibronectin, and β-actin shown by a representative blot from three independent experiments.

When cell morphology was examined, PC3 cells expressing WIF1 were more compact and adherent to adjacent cells than PC3 cells expressing PcDNA3.1, as seen in Figure 3A. This change to a more "adhesive" cellular morphology resembles a transition from a fibroblastic to an epithelial appearance. Furthermore, Figure 3B shows that PC3 cells transfected with WIF1 but not those transfected with PcDNA3.1 vector control exhibit intense staining of E-cadherin along the entire cell-cell contact region among neighboring cells and weaker staining in the contact-free borders.

Consistent with the changes in morphology, ectopic expression of WIF1 in PC3 cells results in a dramatic increase in the protein levels of E-cadherin and keratin-8 and a decrease in N-cadherin, vimentin, and fibronectin (Figure 3C). Together, these results suggest that WIF1 expression causes a reversal of EMT.

### The modulation of EMT markers by WIF1 expression is associated with down-regulation of Slug/Twist expression in PC3 cells

**Figure 4:**
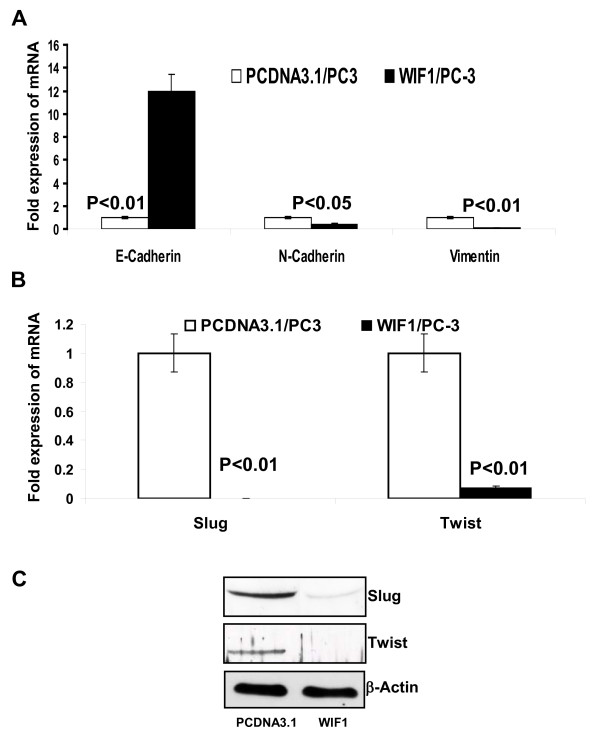
**The modulation of E-cadherin, N-cadherin, and vimentin mRNA levels by WIF1 transfection is associated with its down-regulation of Slug, and Twist expression**. **A & B**, quantitative real-time PCR analysis of E-cadherin, N-cadherin, vimentin, Slug, and Twist mRNA in the transfectants. Gene expression is presented as fold increase in Δ Ct compared with PCDNA3.1 vector control transfectants. *Columns*, mean of four independent quantitative real-time PCR experiments; *Bars*, ± SE. **C**, Western blot analysis of expression of Slug, Twist, and β-actin shown by a representative blot from three independent experiments.

Compared to vector control (PcDNA3.1) expression, real-time PCR analysis also shows that WIF1 expression in PC3 cells causes about 12 fold up-regulation of E-cadherin (P < 0.01), and leads to down-regulation of N-cadherin and Vimentin mRNA expression by 60% and 87%, respectively (Ps < 0.05 and 0.01, respectively) (Figure 4A). This result suggests that the effect of WIF1 on the expression of epithelial and mesenchymal markers in PC3 cells may be associated with transcriptional regulation.

Embroynic transcriptional factors including Slug, Snail, and Twist are master regulators of EMT and repressors for *E-cadherin *gene transcription [35]. In addition, Wnt signaling has been reported to cause up-regulation of the expression of Slug and Twist [36,37]. Figure 4B shows that inhibition of Wnt signaling by WIF1 expression markedly decreases the mRNA expression of Slug and Twist by about 99.9% and 93%, respectively (Ps < 0.01). Consistently, Figure 4C demonstrates that the protein levels of Slug and Twist are also decreased by WIF1 expression. Taken together, our results suggest that the WIF1 induced reversal of EMT in PC3 cells is associated with inhibition of Wnt signaling leading to down-regulation of transcriptional factors *Slug/Twist *and up-regulation of *E-cadherin*.

### WIF1 suppresses cellular motility, and decreases the invasive capacity of PC3 cells via down-regulation of matrix metalloproteinase-2 and -9 activities

**Figure 5:**
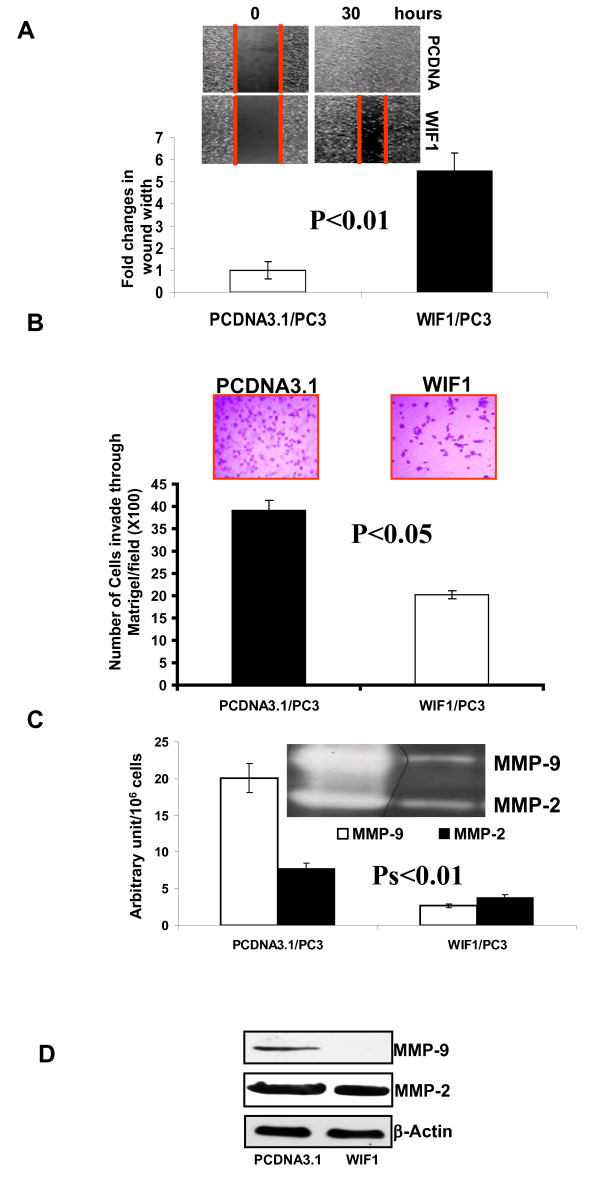
**Ectopic expression of WIF1 in PC3 cells results in a reduction of cell migratory and invasive capacity and a decrease in MMP-2 and-9 expression and activities**. **A**, representative photomicrographs of scratch wounds at 0 and 30 h after wounds were made. Quantitative measurement of wound gaps by Photoshop software showed a reduced cellular motility in the WIF1-transfected PC3 cells compared with vector control cells. *Columns*, mean fold changes of wound width in WIF1 versus vector control-transfected PC3 cells; *bars*, SD. Experiments were replicated thrice. **B**, cells were applied to the upper surface of a Matrigel-coated membrane. After incubation for 48 hours, the upper surface of the membrane was scrubbed free of cells; the membrane was fixed, stained, and photographed. Representative pictures were taken from the lower surface of four independent membranes at ×100 magnification. Invasion index was calculated by adjusting cellular motility as described in detail in Materials and Methods. *Columns*, mean of invasion index calculated from four independent membranes; *bars*, SE. **C**, MMP-2 and MMP-9 activities in the conditioned medium from the transfectants were assayed by zymography as described in Materials and Methods. Semiquantitative comparison of MMP activities between PCDNA3.1 vector control and WIF1-transfected cells was done by densitometry. *Columns*, mean from three independent experiments; *bars*, SE. **D**, Western blot analysis of MMP-2 and MMP-9 expression. Representative blots were from three independent experiments. β-Actin serves as loading control.

Based on the effects of WIF1 on EMT reversal, we next examined the effect of WIF1 expression on migration of PC3 cells using a wound healing assay. Figures 5A shows that WIF1 transfected PC3 cells exhibited about 5.5 fold slower migration into the wounded area, measured by width of the gap, when compared with vector control PcDNA3.1 transfected PC3 cells (P < 0.01).

We also examined the *in vitro *invasiveness of PC3 cells expressing WIF1, or vector control in a Matrigel invasion assay. The capacity of these cells to invade through a Matrigel-coated membrane was expressed as average number of migrated cells on the lower surfaces of triplicate membranes and adjusted by cell motility. Cell motility was measured by average number of cells migrating through a control, uncoated insert. Number of cells on each membrane was averaged from 10 fields (×100). WIF1-transfected cells exhibited a significant decrease in invasive capacity (by 49%) compared with control cells (Student's t test, P < 0.05; Figure. 5B).

MMP-2 and -9 play an important role in cell-matrix interaction and tumor invasion in PCa [38] and Wnt signaling has been reported to regulate the expression of MMPs [39,40]. We therefore examined the effect of WIF1 on MMP-2 and MMP-9 activities and expression. Zymography shows that ectopic expression of WIF1 in PC3 cells resulted in decreased activities of both MMP-2 (lower band) and MMP-9 (upper band) in conditioned medium (Figure 5C). The decrease in MMP-2 and-9 activities was correlated with lower MMP-2 and MMP-9 protein levels (Figure 5D). MMP-2 and MMP-9 activities were also quantified by densitometric analysis and adjusted by total number of cells in each culture. Compared with vector control transfectants, WIF1 transfectants showed a decrease in MMP-2 and -9 activities by 87% and 51%, respectively (Figure. 5C; Student's t test, Ps < 0.01).

### WIF1 inhibits in vivo tumor growth of PC3 cells in a xenograft mouse model, induces the expression of E-cadherin and karatin-18 and decreases the expression of Vimentin in tumor tissues

**Figure 6:**
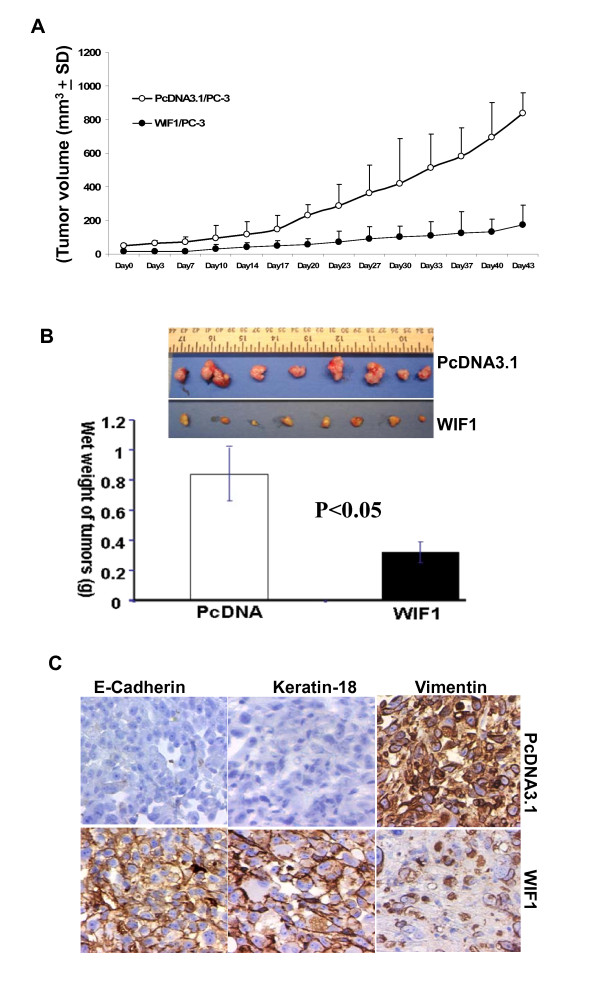
**Ectopic expression of WIF1 in PC3 cells inhibits tumor growth in a xenograft model**. PC3 cells (2 × 106) stably transfected with vector control or WIF1 were injected into the left flank of NCR-*nu/nu *(nude) mice. ***A***, points, mean tumor volume (each group contains 13 mice); *Bars*, SD. ***B***, at the termination of the study on day 48, tumors were excised from each mouse in different groups and weighed. Wet weight of tumors is represented as means of 13 tumors from individual mouse in each group. ***P ***< 0.05 at the termination of the experiments; *Bars*, ± SD. ***C***, Immunohistochemical staining of ectopic E-cadherin, Keratin-18 and vimentin expression in harvested mouse tumor samples.

Figure 6A shows that the ectopic expression of WIF1 in PC3 cells resulted in a significant decrease in the growth rate of tumors compared to control (ANOVA, ***P ***< 0.01). The wet tumor weights were 0.84 ± 0.256 g in the control group and 0.314 ± 0.18 g in the WIF1- transfectants group (Figure 6B; n = 13, mean ± SD; ***P ***< 0.05, Student's t test). Ectopic WIF1 expression attenuated tumor growth by approximately 63%.

Immunohistochemical analysis shows that tumor sections from the WIF1 transfected PC3 cell line exhibited a marked increase in E-cadherin and Keratin-18 while tumor sections from vector control cell line revealed no or very weak staining (Figure 6C). In contrast, the density of Vimentin staining was significantly decreased in the WIF1 tumors compared to control tumors (Figure 6C).

## Discussion

Bone metastases occur in approximately 70% of patients with advanced PCa, causing severe morbidity and hospitalization [34]. WIF1 has been shown to play an important functional role in the development of prostate and bone [28-31]. Thus, the loss or down-regulation of WIF1 expression in PCa may have pathophysiological consequences and, therefore, contribute to PCa development and its metastasis to bone. Although WIF1 has been shown to act as a tumor suppressor, inhibiting cancer cell proliferation in many cancers [17-27], little is known about its potential effect on metastatic tumors and the process of tumor metastasis. We reported here that WIF1 promoter was hypermethylated in the majority of PCa cell lines, leading to the absence of WIF1 mRNA expression in these cell lines. Re-expression of WIF1 in a bone metastatic PCa cell line, PC3, resulted in an *in vivo *inhibition of tumor growth and a decreased capacity of cell migration and invasion. This suggests that the loss of WIF1 in PCa may contribute to its metastatic potential. WIF1's mechanism of action is associated with a reversal of EMT process *via *down-regulation of E-cadherin expression, up-regulation of N-cadherin expression, and decreased activity of MMP-2 and -9.

The EMT process was initially described in the context of embryonic development and has recently been thought to regulate invasive behavior of epithelial cancer cells at the tumor-stromal interface [35]. EMT is characterized by increased migratory features, decreased epithelial cell adhesion, loss of cytoskeleton components and acquisition of mesenchymal components [35]. A hallmark of EMT is the loss of E-cadherin expression [35]. Embryonic transcription factors (The Slug/Snail, Ets, and Twist) are transcriptional repressors of E-cadherin and the converging points for EMT-related pathways [35]. At present, there are a very few reports that agents can reverse EMT process in metastatic cancer cells. We showed that re-expression of WIF1 in PC3 cells changed cell morphology from spindle-shaped to a cobblestone-like monolayer, increased expression of epithelial markers (i.e. E-Cadherin and Keratin-8 and -18) and decreased expression of mesenchymal markers (i.e. N-cadherin and Vimentin) both *in vitro *cell cultures and in *in vivo *tumor tissues. The increased expression of E-cadherin in the PC3 cell with over-expression of WIF1 was associated with down-regulation of the mRNA and protein levels of two transcriptional repressors: Slug and Twist. Similarly, in a recent study Xie et al [41] demonstrated that in metastatic PCa cells restoring the expression of Disabled homolog 2 interacting protein (DAB2IP), a Ras GTPase-acting protein, leads to a reversal of EMT by interacting with PP2A and GSK-3β and decreasing nuclear β-catenin accumulation and its transcriptional activity. In an inducible FGFR1 prostate mouse model that exhibited a highly synchronous, step-wise progression to adenocarcinoma, Acevedo et al [42] reported that the increase in Frizzled-4 receptor levels and the decrease in WIF1 levels were related to EMT mediated cancer progression. In addition, we have shown that ectopic expression of a dominant-negative LRP5 in PC3 cells reversed EMT [32]. Taken together, given LRP5 is a co-receptor of Frizzled-4 receptor [43], these studies suggest that the Frizzled-4/LRP5 mediated β-catenin/TCF4 pathway may participate in regulation of the EMT process in PCa progression and that WIF1 may interfere with Wnts/Frizzled-4 or LRP5 interaction leading to a reversal of EMT. Therefore, further studies are in progress to determine which Wnts or Frizzled-4 ligands WIF1 could bind to in order to inhibit the interaction between Frizzled/LRP5 and to reverse EMT.

The antiproliferation effect of WIF1 has been consistently reported in a wide variety of other cancer cell lines [17-26]. In prostate cancer, Ohigashi et al [27] reported that WIF1 overexpression only enhanced Paclitaxel-induced apoptosis in PC3 Cells. However, we were unable to detect any significant changes in cell proliferation and apoptosis by WIF1 overexpression alone in PC3 cells (data not shown). It is possible that the selection process of establishing stable clones may attenuate the antiproliferative effect of WIF1. However, we did show that WIF1 overexpression significantly inhibits *in vivo *tumor growth of PC3 cells in a xenograft model. Thus, it is reasonable to speculate that WIF1 may have an anti-angiogenesis effect on tumor growth. Hu et al [44] recently reported that the inhibition of tumor growth by adenoviral delivery of WIF1-human IgG1 Fc fragment fusion protein in hepatocellular carcinoma mouse xenograft models is associated with reduced microvessel density, decreased expression of vascular endothelial growth factor, decreased stromal cell-derived factor-1 and increased apoptosis. Emerging evidence also suggest that Wnt signaling plays an important role in vascular development [45]. However, the potential effects and mechanism of WIF1 on angiogenesis are largely unexplored. Therefore, it is important to study whether WIF1 will have an anti-angiogenesis effect on PCa. It is also important to understand the potential molecular mechanisms of WIF1's effect on angiogenesis.

## Conclusions

The frequent absence of WIF1 expression in PCa cell lines is associated with its promoter hypermethylation. Restoring WIF1 expression in WIF1 deficient PCa cells resulted in a decreased cellular capacity of migration and invasion, which was associated with a reversal of EMT. In addition, WIF1 restoration inhibited the *in vivo *growth of PCa cancer cells in a xenograft model. Together, these results suggest that the potential of WIF1 as an anti- invasiveness agent alone or in combination with other agents deserves further study in the treatment of castration-resistant PCa.

## List of abbreviations

DAB2IP: Disabled homolog 2 interacting protein; Dkk: Dickkopf; EMT: Epithelial-to-Mesenchymal Transition; FBS: fetal bovine serum; LEF1: lymphoid enhancer binding factor 1; LRP5/6: Lipoprotein Receptor-Related Protein 5/6; MMP: metalloproteinase; PCa: Prostate cancer; RT-PCR: reverse-transcription polymerase chain reaction; sFRP: secreted Frizzled-related protein family; TCF-4: transcription factor 4; WIF1: Wnt inhibitory factor 1; Wnt: wingless-type

## Competing interests

The authors declare that they have no competing interests.

## Authors' contributions

Study concept and design: XZ, BH, AS, AD; acquisition of data: DY, JX, YG, YT, SL, ZL, UW, NAG, WA; analysis and interpretation of data: XZ, DY, YT, YG; drafting and critical revision of the manuscript: XZ, DY, SG, PM; of the manuscript for important intellectual content JD, PEH, HF; statistical analysis: XZ; obtained funding: XZ; technical and material support: XZ, YG; study supervision: XZ, BH. All authors have read and approved the final manuscript.
